# Efficacy of the new therapeutic approach in curing malignant neoplasms on the model of human glioblastoma

**DOI:** 10.20892/j.issn.2095-3941.2020.0511

**Published:** 2021-08-15

**Authors:** Evgeniya V. Dolgova, Oleg M. Andrushkevich, Polina E. Kisaretova, Anastasia S. Proskurina, Genrikh S. Ritter, Tatyana D. Dubatolova, Margarita V. Romanenko, Oleg S. Taranov, Yaroslav R. Efremov, Evgeniy L. Zavyalov, Alexandr V. Romaschenko, Sergey V. Mishinov, Svetlana S. Kirikovich, Evgeniy V. Levites, Ekaterina A. Potter, Alexandr A. Ostanin, Elena R. Chernykh, Stanislav Yu. Roshchin, Anatoliy V. Bervitskiy, Galina I. Moysak, Jamil A. Rzaev, Sergey S. Bogachev

**Affiliations:** 1 Institute of Cytology and Genetics SB RAS, Novosibirsk 630090, Russia; 2 A.I. Evdokimov Moscow State University of Medicine and Dentistry, Moscow 127473, Russia; 3 Institute of Molecular and Cellular Biology, Novosibirsk 630090, Russia; 4 Novosibirsk State University, Novosibirsk 630090, Russia; 5 The State Research Center of Virology and Biotechnology “Vector”, Koltsovo, Novosibirsk 630559, Russia; 6 First Department of Neurosurgery, Ya. L. Tsivian Novosibirsk Research Institute of Traumatology and Orthopaedics, Novosibirsk 630091, Russia; 7 Institute of Fundamental and Clinical immunology, Novosibirsk 630099, Russia; 8 Sklifosovsky Research Institute of Emergency Medicine, Moscow 129010, Russia; 9 Federal Center of Neurosurgery, Novosibirsk 630048, Russia

**Keywords:** Glioblastoma, U87 cell line, mytomycin C, cancer stem cells, TAMRA

## Abstract

**Objective:**

Glioma is a highly invasive tumor, frequently disposed in essential areas of the brain, which makes its surgical excision extremely difficult; meanwhile adjuvant therapy remains quite ineffective.

**Methods:**

In the current report, a new therapeutic approach in curing malignant neoplasms has been performed on the U87 human glioblastoma model. This approach, termed “Karanahan”, is aimed at the eradication of cancer stem cells (CSCs), which were recently shown to be capable of internalizing fragments of extracellular double-stranded DNA. After being internalized, these fragments interfere in the process of repairing interstrand cross-links caused by exposure to appropriate cytostatics, and such an interference results either in elimination of CSCs or in the loss of their tumorigenic potency. Implementation of the approach requires a scheduled administration of cytostatic and complex composite double-stranded DNA preparation.

**Results:**

U87 cells treated *in vitro* in accordance with the Karanahan approach completely lost their tumorigenicity and produced no grafts upon intracerebral transplantation into immunodeficient mice. In SCID mice with developed subcutaneous grafts, the treatment resulted in reliable slowing down of tumor growth rate (*P* < 0.05). In the experiment with intracerebral transplantation of U87 cells followed by surgical excision of the developed graft and subsequent therapeutic treatment, the Karanahan approach was shown to reliably slow down the tumor growth rate and increase the median survival of the mice twofold relative to the control.

**Conclusions:**

The effectiveness of the Karanahan approach has been demonstrated both *in vitro* and *in vivo* in treating developed subcutaneous grafts as well as orthotopic grafts after surgical excision of the tumor.

## Introduction

Despite the relatively low incidence, central nervous system (CNS) malignancies are of high lethal outcome risk: the median survival after diagnosis is approximately 10–15 months^1,2^. A significant portion of diagnosed CNS tumors occur in children and adolescents, and the incidence in children younger than 15 years is increasing by 1.8% annually^3,4^.

CNS neoplasms occur in a variety of spinal cord and brain compartments. Gliomas account for an average of 24% of all brain tumors and are the second most common brain tumor in adults^4^. The average survival time of patients with anaplastic astrocytoma and glioblastoma is 15.2 and 6.9 months, respectively^5^. According to the Cancer Research UK data, the 5-year survival of patients with anaplastic astrocytoma is 25% of cases, whereas for patients with glioblastoma, this indicator is only 6%. Hence, the development of new approaches in the brain tumors diagnosis and treatment seems to be one of the most urgent tasks of experimental oncology.

Surgical excision of the tumor bulk followed by a course of chemo/radiotherapy is the most common approach in treating gliomas. As a chemotherapeutic agent, temozolomide (TMZ) is among the most recommended preparations. TMZ belongs to the family of alkylating cytostatics capable of passing through the blood–brain barrier (BBB)^6,7^. The use of TMZ extends the median survival of patients up to 14.6 months after diagnosis^1^. Such a low therapeutic efficacy is due to a number of factors ensuring the resistance of gliomas to treatment. First, the structure of these tumors (infiltrating growth and lack of a capsule) does not allow the perfect excision of tumor bulk, and the remaining cells can produce a relapse. Second, gliomas are usually very hypoxic tumors. Low oxygen content in the tumor bulk drastically decreases the efficacy of chemo/radiotherapy^7^. Third, passing through the BBB seriously reduces the potency of alkylating agents. In addition, there is a variety of mutations, negating the potency of anti-tumor agents^8^. However, the main source of resistance of glioblastomas to existing therapeutic schemes is the presence of cancer stem cells (CSCs), which have incredible survivability and proliferative capacities^9–11^.

Glioma CSCs display properties similar to those of neural stem cells: indefinite self-renewal, formation of neurospheres, expression of certain markers (CD133, CD15, A2B5, CD44), differentiating and migratory capacities, and very specific microenvironment^5,12^. Glioma CSCs are believed to constitute the subpopulation resistant to the effects of cytostatics and gamma radiation^13^. Finally, cells of this type exert immunosuppressive properties^14^.

Detection of CSCs, the main target in the bulk of tumor cells, is the primary objective, which is inevitably followed by the issue of killing these cells in their native environment. The native properties of CSCs, which include proliferative self-sufficiency, enhanced motility, innate drug resistance, independence on stem niches environment, capability of “blebbishielding” (formation of viable cells by fusion of apoptotic blebs), and epigenetic plasticity (failure of one signalling pathway results in compensatory activation of an alternative one, causing both the overall survival and increased aggressiveness of the progeny) with presumed capability of reverting a committed phenotype to a stem-like one under stress conditions, make them extremely resistant to a variety of external effects^5,7,15,16^.

Currently, the possibility to affect certain molecular targets in CSCs of glioblastomas is being investigated^17–20^. However, despite tremendous efforts, no effective therapeutic scheme allowing the cure of glioma patients, especially those with glioblastomas, or at least significantly increasing their relapse-free survival has been developed yet. In this regard, the search for new markers and development of fundamentally new approaches aimed at removing glioma CSCs remains a primary objective in experimental and clinical oncology.

Until now, the search for a universal marker specific for CSCs of gliomas has been extremely straightforward. It was based on the idea that there should be a certain unique factor either on the surface of such cells or in their internal compartments that can be physically isolated and marked with any of currently known ways^5,12,21–24^.

Previously, we have described the marker property of a variety of poorly differentiated cells, including CSCs, which manifests itself as the capability of native internalization of extracellular double-stranded DNA (dsDNA) fragments. To detect CSCs by this capability, a TAMRA-labeled DNA probe was used^25,26^. Cells internalizing the TAMRA-DNA probe are hereinafter referred to as TAMRA+ cells. Using this new marker property, CSCs were detected in human glioblastomas, and their amount directly correlated with the grade of tumor^11^. We have also discovered that internalized dsDNA fragments interfere in the process of repairing interstrand cross-links (ICLs), which occur upon exposure to cross-linking agents. Based on these findings, we have developed the approach allowing the synchronization and accumulation of CSCs in the certain cell cycle phase when they are very sensitive to treatment. Further, this approach, termed “Karanahan” or “3 + 1”, has been converted into the full-scale universal CSCs eradication technology^27–31^.

Currently, we are reporting on the applicability of the aforementioned approach to the disruption of U87 cell tumorigenicity, treatment of their subcutaneous grafts, and cure of orthotopic grafts after surgical excision of the tumor.

## Materials and methods

### Obtaining the TAMRA-labeled DNA probe and the complex composite dsDNA preparation

Human *Alu* repeat fragment was labeled with TAMRA dye using PCR as described by Dolgova et al.^26^. Complex composite dsDNA preparation (“DNAmix” throughout) was prepared as described by Potter et al.^29^.

### Detecting TAMRA+/CD133+ cells

Cells were seeded in 3.5 cm Petri dishes. Adherent cells were washed with phosphate-buffered saline (PBS) and then added with serum-free medium supplemented with 0.5 µg/mL *Alu*-TAMRA DNA and incubated for 20 min. Incubated cells were washed with PBS and placed in medium with serum for 5 min, washed once again, and further incubated with APC-conjugated CD133-specific antibodies (Miltenyi Biotec, Bergisch Gladbach, Germany) (1:150) for 20 min at the room temperature. Then, Hoechst nuclear dye (Thermo Fisher Scientific, Waltham, MA, USA) was added and incubated for another 15 min. Cells were assayed on Axio Observer Z1 fluorescence microscope or LSM 780 NLO (Zeiss, Jena, Germany) confocal fluorescence microscope with ZEN software (Zeiss). Calculation of TAMRA+ cells was performed according to the Order of the Ministry of Health of the Russian Federation dated March 21, 2003, “Improvement of anti-tuberculosis measures in the Russian Federation”, paragraph #11.4 “Microscopic examination procedure”. For each sample, 2,000–4,000 cells were assayed. Alternatively, the specimens were FACS assayed using FACSAria III cytometer and FACSDiVa software (Becton Dickinson, Franklin Lakes, NJ, USA). APC-conjugated mouse IgG1 antibodies (Miltenyi Biotec, Bergisch Gladbach, Germany) were used for baseline detection.

### Experimental animals

We used 6- to 7-week-old SCID mice housed in the SPF Animal Facility (Institute of Cytology and Genetics SB RAS). Animals were kept as single-sex family groups of 2–5 individuals in OptiMice IVC cages (Animal Care Systems, Centennial, CO, USA) in special cleanrooms with HEPA14-filtered incoming air with temperature of 22 ± 2 °C, humidity of 45% ± 15%, and light/darkness regimen of 14/10 h. Ssniff food (Soest, Germany) and water enriched with the Severyanka (St. Petersburg, Russia) mineral mixture were provided to animals at libitum. All experiments with animals were conducted in strict compliance with the principles of humanity in accordance with the European Community Council Directives (86/609/EEC) and were approved by the Animal Care and Use Committee of the Institute of Cytology and Genetics (ICG) SB RAS.

### Subcutaneous grafting U87 cells into SCID mice

Prior to grafting, U87 glioblastoma cells (provided by the cell repository ICG SB RAS) were cultivated for 5–7 passages in Dulbecco’s modified Eagle medium (DMEM; Gibco, Carlsbad, CA, USA) supplemented with 10% FBS (Invitrogen). For grafting, cells were detached with trypsin/EDTA solution and sedimented at 400 *g* for 5 min. The obtained pellet was thoroughly resuspended in serum-free medium up to the required concentration. To obtain a solid form of U87 glioma, mice were subcutaneously grafted into the interscapular area with 6.3 × 10^6^ cells in 100 µL of serum-free DMEM. Tumor growth was monitored regularly every 2–3 days. As soon as characteristic subcutaneous nodules appeared, tumors began to be measured.

### Treatment of subcutaneous U87 glioblastoma grafts according to the Karanahan approach

U87 cells were subcutaneously engrafted into SCID mice as described above. Fifteen days later, animals with developed grafts were selected and treated in accordance with the following scheme: cyclophosphamide (CP) was administered intraperitoneally in doses of 70 mg/kg at hours 0, 36, and 72 and at day 7 after the start of therapy; the composite dsDNA preparation (DNAmix) in doses of 0.5 mg per mouse was administered intratumorally into three random points of the tumor node 18 h after each CP injection. Tumors were measured every 3 days, and their volume was calculated by the formula length × width^2^ × 0.5^32^.

### Determination of the DNA repair cycle duration by the comet assay

Suspension of human glioblastoma cells (3 × 10^5^ cells) was incubated in 1 mL DMEM supplemented with 20 µg/mL of mitomycin C (MMC) (Sigma-Aldrich, St. Louis, MO, USA) at 37 °C for 1 h. After incubation, cells were sedimented, washed with medium supplemented with 10% serum, and resuspended in the same medium and seeded on a 24-well plate. Every 6 h, cells were sampled and fixed in agarose blocks (Low Melt Ultra-Pure DNA Grade Agarose; Bio-Rad, Hercules, CA, USA) at 4 °C. After solidification, blocks were transferred into 500 mM EDTA. Prior to electrophoresis, blocks were washed in 1 × TE, transferred into lysis buffer [1% *N*-lauroylsarcosine (Biophoretics, Sparks, NV, USA), 50 mM EDTA, 1 mg/mL proteinase K] and lysed at 50 °C for 15 min. Electrophoresis was conducted at 0.75 V/cm for 30 min in 1 × TAE buffer supplemented with ethidium bromide. After electrophoresis, the gel was dried and assayed using Axio Imager (Zeiss) fluorescence microscope and ISIS software version 5.4.9 (MetaSystems, Altlussheim, Germany). Double-strand breaks (DSBs) were assessed by the “Tail Moment”™ (TM = tail length × DNA percentage in the tail) in the CASP software (CASP, Wroclaw, Poland).

### Cell cycle assay

Glioblastoma cells were thrice exposed to MMC (20 µg/mL) every 36 h. Cells were sampled before and 18, 54, and 90 h later as well as on days 6–9 after the first exposure. Sampled cells were fixed with 50% methanol and stored at 4 °C until the assay. Immediately prior to assay, equal numbers of cells from each sample were sedimented at 400 *g* for 5 min, washed with PBS, resuspended in staining solution supplemented with propidium iodide (Sigma-Aldrich) and RNase, and incubated at 37 °C for 30 min. Cell cycle assay was conducted for all samples simultaneously on FACSAria III (Becton Dickinson).

### Exposure of glioblastoma cells to a cytostatic and DNAmix *in vitro* and counting TAMRA+ cells

Adherent cells were washed with PBS and added with serum-free medium supplemented with either MMC (5 or 20 µg/mL) or DNAmix (0.5 µg/mL). Cells were incubated for 1 h with MMC and for 40 min with DNAmix. After incubation, cells were washed with PBS and added with DMEM supplemented with 10% FBS. Upon reaching the appropriate experiment check point, the corresponding Petri dish was taken to count the TAMRA+ cells percentage, as described above. After this, cells were detached with trypsin/EDTA, and their absolute number (N) was assessed in the Goryaev chamber. The absolute number of TAMRA+ cells (X) was calculated by the formula X = N × (% TAMRA+ cells)/100.

### Intracerebral grafting of human glioblastoma cells into SCID mice

To estimate the grafts’ development rate, U87 glioblastoma cells pretreated with the cytostatic and DNAmix preparation *in vitro* were orthotopically grafted into SCID mice. Five microliters of cell suspension (3 × 10^5^ cells total) were intracerebrally injected into the left hemisphere of experimental animals.

For experiments on surgical excision of developed grafts, each animal (15 mice total) was subcortically injected with 3 µL of U87 cell suspension (3 × 10^5^ cells total) in DMEM into the parietal lobe of the brain left hemisphere to a depth of 2.5–3 mm (counting from the skin surface).

### Monitoring the development and growth rates of U87 xenografts in experimental animals

Graft development and growth rate were monitored on the BioSpec 117/16USR NMR tomographic scanner (Bruker, Karlsruhe, Germany) using T2-weighted technique on day 24 after the grafting procedure. All examinations were performed in axial, coronary, and sagittal projections.

### Neurosurgical excision of the developed tumor

Surgical procedures were performed at the Federal Neurosurgery Center, Novosibirsk. All procedures were carried out in accordance with the data of NMR tomography regarding tumor localization and accessibility. During the postsurgical period (5 days) prior to chemotherapy, 80 µg of gentamicin was administered daily per mouse.

We have designed the strategy for surgical excision of intracerebral grafts in mice, which is as close as possible to real clinical conditions. Steps and corresponding techniques of the neurosurgical intervention are illustrated in **Supplementary Figure S1**. Anesthetic support comprised XylaVet (Pharmamagist Ltd, Hungary) (120 µg per mouse with average weight of 21 g) and Telazol (Zoetis, Parsippany, NJ, USA) (300 µg per mouse) adjusted up to 100 µL with a physiological saline solution and injected intramuscularly. The resulting duration of anesthesia was about 35–40 min. Antibiotic prophylaxis consisted of 80 µg gentamicin per mouse, administered intraperitoneally or subcutaneously. Prior to the surgical procedure, animals were fixed on the tablet and covered with a sterile cover. To prevent antiseptic agents from getting into the eyes of the animal as well as potential development of ulcerative keratitis, blepharorrhaphy was performed on both sides by applying single sutures (**Supplementary Figure S1B1**). The incision area was pretreated with an alcohol solution of chlorhexidine and then injected with a 1% Novocaine (Velpharm, Moscow, Russia) solution (**Supplementary Figure S1B2**). After that, the soft tissues of the head were linearly incised along the midline (**Supplementary Figure S1B3**). The scalp was diverted by four holding stitches imposed on the “mosquitoes”. The cranial bone was exposed after subperiosteal dissection (**Supplementary Figure S1B4**). A craniotomy window measuring 5 × 5 mm in the projection of the developed tumor (left parietal–occipital area) was made to the depth of the *lamina vitrea* using the “Stryker” tool and a diamond drill with a tip of 2 mm diameter (**Supplementary Figure S1B5**). The location of the neoplasm in the mouse brain was assessed using preoperative NMR scanning. To prevent the heating of bones and underlying tissues as well as to avoid the fusion of bone shavings with the drill surface, the surgical area was irrigated with a warm sterile physiological saline solution during the drilling. The surgical wound was also washed with a warm (36–37 °C) sterile physiological saline solution to remove residual bone shavings. Remnants of the *lamina vitrea* were removed using tweezers and a bone elevator. Sterile covers moistened with physiological saline solution were put around the surgical wound (**Supplementary Figure S1B6**). Corticotomy and subsequent resection of the tumor in the parietal lobe were performed with an insulin syringe (**Supplementary Figure S1B7 and S1B8**). As the next step, thorough hemostasis was performed. Plastic surgery of the craniotomy defect in the bone was not performed. The wound was sutured with a continuous twining seam on the skin using 6/0 Vicryl, a dissolvable suture material on an atraumatic needle (**Supplementary Figure S1B9**). At the final stage, sutures and surgical wound area were treated with an alcohol solution of chlorhexidine, and those imposed during blepharorrhaphy were removed (**Supplementary Figure S1B10**).

### Statistical processing of data

For statistical processing of data, Statistica version 10 (StatSoft, Tulsa, OK, USA) was used. Statistical routines used for each particular experiment are indicated in the figure legends. In the plots, either the average value ± 99% confidence interval (CI) or the median ± standard error of the mean (*n* = 6–7) are given. Confidence was estimated with either Student’s t-test (in cases of Gaussian distribution) or Mann–Whitney U-test (in cases of non-Gaussian distribution).

## Results

The Karanahan, or 3 + 1 approach is based on three main findings made in the Laboratory of Induced Cellular Processes, ICG SB RAS (Russia):

1. Discovery of the universal capability of poorly differentiated cells, including CSCs, to natively internalize the fragments of extracellular dsDNA, including those labeled with TAMRA fluorescent dye.
2. Discovery of the interference of these internalized DNA fragments in the process of repairing DNA ICLs that results either in the elimination of such cells or in the loss of their tumorigenic potency.
3. Discovery of the technique allowing the synchronization of tumor cells, including CSCs, in the certain cell cycle phase, when they become susceptible to the terminal treatment, resulting in elimination of CSCs as the main source of tumor existence.

The approach comprises the following procedures:

1. Detection of poorly differentiated CSCs in each particular tumor by their capability of internalizing the TAMRA-labeled DNA probe.
2. Finding the time profile of the ICLs repair process after exposure to cross-linking cytostatic agents.
3. Synchronization of proliferating cells, including CSCs, by triple exposure to a cytostatic in accordance with the determined length of ICLs repair process, followed by detecting the time when these cells exit the cell cycle arrest, accumulate in G2/M and synchronously transit into G1, becoming vulnerable to the terminal exposure to a cytostatic.
4. Administration of the DNAmix preparation at the point demarcating the phases of nucleotide excision repair (NER) and homologous recombination (HR). One of the components of this preparation interferes in the NER, whereas another component interferes in the HR, causing CSCs either to completely lose their capability of surviving the “therapeutic strike” or at least to forfeit their tumorigenic properties. Moreover, this treatment induces a rapid large-scale lysis of the bulk of committed tumor cells, especially those actively proliferating. The referred DNAmix preparation was previously developed in the Laboratory of Induced Cellular Processes, ICG SB RAS (Russia)^27,29^.

Briefly, the schedule of cytostatic and DNAmix administration within the frames of the Karanahan approach is determined as follows: a cross-linking cytostatic is administered three times exactly at the times of maximal decrease in the number of DSBs with the interval corresponding to the duration of DNA repair in tumor cells; the fourth cytostatic administration is to be performed when cells are synchronized in the G2/M phases of the cell cycle due to the previous three exposures; the composite dsDNA preparation is to be administered after every exposure to a cross-linking cytostatic exactly at the time of maximal accumulation of DSBs in the exposed cells, which indicates the demarcation of NER and HR phases of the DNA repair process. The first three exposures result in the large-scale caspase-activated DNase-dependent apoptosis of committed and partially stem-like tumor cells, as we have demonstrated in Krebs-2 and some other experimental tumors. The fourth terminal exposure is supposed to completely eliminate the remaining tumor cells, including the TAMRA+ CSCs, observed in the Krebs-2 model^28,31,33^.

All the aforementioned procedures were completed for U87 cells, and the results obtained were used in further *in vitro* experiments and *in vivo* treatment of grafted mice.

### Assessment of the basic indicative Karanahan parameters for the U87 human glioblastoma cell line

#### Assessing the content of TAMRA+/CD133+ cells in U87 human glioblastoma cell line in vitro

**Figure 1:**
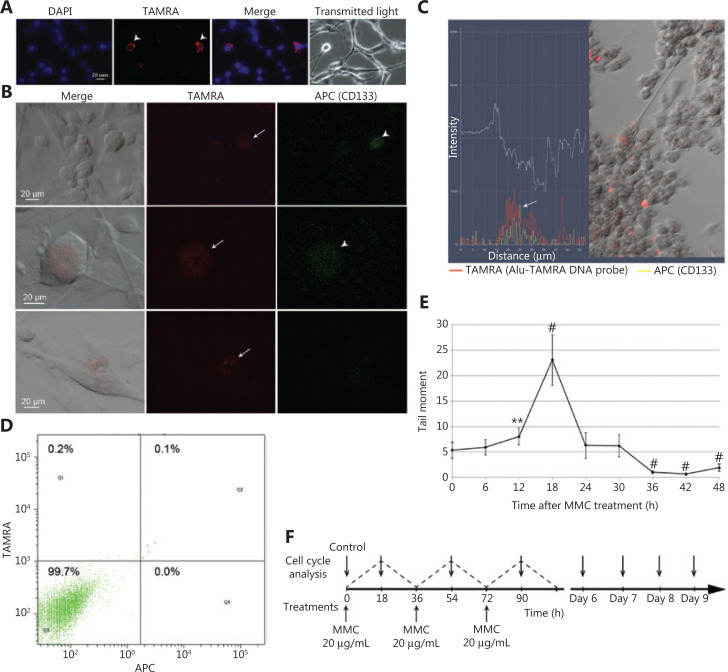
The basic Karanahan approach parameters and treatment schedule. (A) TAMRA+ cells in *in vitro* U87 cell culture. (B) Cross-examination of U87 cells with regard to their ability both to bind antibodies to the surface marker CD133 and to internalize the TAMRA-DNA probe. Arrows indicate TAMRA+ cells, arrowheads indicate CD133+ cells. (C) Combined fluorescence profile along the vector crossing the single U87 cell [result of processing the image obtained with LSM 780 NLO confocal microscope (Zeiss) in Zen software package]. Arrow indicates the site of colocalization of TAMRA-DNA and anti-CD133 antibodies signals. (D) FACS assay of the content of TAMRA+/CD133+ cells in *in vitro* U87 cell culture. DAPI, chromatin staining; TAMRA, fluorescence of the TAMRA-labeled DNA probe; APC, anti-CD133 antibodies. (E) Profile of DNA DSB occurrence in U87 cells exposed to MMC. The number of DSBs was determined as the “tail moment” during the comet assay. The average value ± 99% confidence intervals are given; confidence was estimated relative to the “zero point” using the Student’s criterion in the Statistica 10 software package, **P < 0.01, ^#^*P* < 0.001. (F) Schedule for MMC treatments (three rounds every 36 h) as well as for the cell cycle assays.

U87 is the standard cellular model used for investigating glioblastomas. These cells demonstrate exponential adherent growth in DMEM supplemented with 10% fetal calf serum (FCS), but in serum-free medium, they form free-floating spheres (unpublished data). Under regular conditions (DMEM + 10% FCS), the adherent U87 culture was shown to contain 0.42% ± 0.26% of TAMRA+ cells (**Figure 1A**).

In all our works related to the characterization of a variety of biological properties of TAMRA+ cells in different experimental tumors and bioptic specimens, we discuss the cross-relation between these cells and cancer stem ones. Thus, in the model of murine Krebs-2 carcinoma, we have demonstrated these cells to possess the main properties of CSCs both in functional and transcriptomic aspects^26,34^. Preliminary results of transcriptome analysis of TAMRA+ cells from the Epstein–Barr virus–induced lymphoblastoid cell line^35^ indicate an elevated expression of genes specific for poorly differentiated cells, including pluripotent ones (data in progress). Being depleted of TAMRA+ cells, this cell line was also shown to be incapable of producing stably growing grafts. The same data were obtained in experiments with human glioblastoma primary cell line, when only floating spheres containing no less than 20% of TAMRA+ cells were tumorigenic, whereas the adherent TAMRA-negative fraction could not produce grafts^26^. This body of evidence presumes the TAMRA+ cells to be related to the subpopulation of CSCs in the reported tumors.

Subpopulations of TAMRA+ cells were also shown to partially or, in some cases, almost completely overlap with the fraction of cells expressing the appropriate markers of stemness in a variety of human breast cancers and glioblastomas^11^. Assuming all the aforementioned, U87 TAMARA+ cells are expected to be positive (at least partially) for CD133 (Prominin-1), the commonly accepted marker of glioblastoma CSCs, and, thus, can be referred to as U87 CSCs.

Experiments on colocalization of these two markers revealed 0.1% of CD133+ cells in the culture of U87 glioblastoma, and all of them were TAMRA+ also. At the same time, the content of TAMRA+ cells in this culture was a bit higher and varied in the range of 0.3%–0.8% (**Figure 1B–1D**). This result confirms the presumed relevance of TAMRA+ cells to the fraction of CSCs.

The presence of TAMRA+/CD133+ cells in the population of U87 glioblastoma cells *in vitro* and their *in vivo* tumorigenicity upon transplantation into immunodeficient mice have allowed the use of this cell line as a reliable and convenient model for the stated purposes of determining the therapeutic scheme and estimating its efficacy in curing this particular glioblastoma.

#### Determination of the DNA ICLs repair time-profile in U87 cells after exposure to MMC in vitro

One of the most critical parameters for the reported technique is the determination of the ICLs repair time pattern. As the main indicator of this process, the rate of DNA DSBs occurrence has been chosen. The process of ICLs repair is a sequence of the following events. Cross-linking cytostatic or its active metabolite diffuses into a cell and intercalates into chromosomal DNA. Alkylating residues interact with nucleobases of both strands inducing interstrand covalent link formation^36^. This formed structure is highly toxic for a cell, and its appearance activates the DNA repair process consisting of two consequent phases: NER and HR. ICLs are mainly being detected during DNA replication in the S phase when replicative forks encounter them. The Mus81–Eme1 nuclease complex hydrolyzes the single-stranded site of one of the newly synthesized DNA molecules^37,38^ that results in the formation of DSB, which can be detected by staining with anti-γH_2_AX antibodies (specific marker for free DSBs) or by the comet assay^39,40^. This event is believed to be the start of the repair cycle. Further, similar events occur with all detected ICLs, which results in the accumulation of DSBs. At the same time, the molecular machinery of NER is activated, initiating the excision of adducts. As soon as NER is completed, the HR phase, which comprises the processing of free double-stranded ends, their annealing with homologous site of the sister chromatid, and final restoration of DNA replication, begins. The disappearance of DSBs, which can easily be detected by the aforementioned approaches, indicates the end of the repair process. Thus, having determined the rate of DSBs occurrence, we determine the duration of the repair process required for the complete restoration of the chromatin integrity, disrupted by the action of cytostatic agent.

The time profile of the DNA repair process in tumor cells is the basis both for scheduling the intermittent administration of cross-linking cytostatic compounds, when each following portion of the compound is administered precisely at the end of repairing lesions caused by the previous treatment, and for determining the appropriate time for concomitant administration of the DNAmix preparation. It also allows to determine the time for the terminal treatment aimed at the most complete elimination of CSCs. It is presumed that, being thrice treated with cross-linking cytostatic agent, tumor cells (those, which survived this process) are either arrested in G1 or accumulated in the early S phases of the cell cycle. Later, such “synchronized” cells more or less synchronously accumulate in the appropriate cell cycle phase (presumably late S, as the most time-consuming part of DNA replication process), when they are most susceptible to the action of cross-linking cytostatics. This moment is supposed to be most suitable for the terminal treatment, allowing the complete curing, as it was shown for Krebs-2 ascites^26,28^. Two cross-linking cytostatic agents, MMC and CP, were used in the reported investigation. Possessing an alkylating capability, MMC molecules form ICLs immediately upon administration, while CP has to be metabolized in the liver to produce the active compound, phosphoramide mustard. ICLs formation has been shown to be completed in 3–6 h post administration, regardless of the chemical peculiarities of the compound used, which, therefore, produce an absolutely insignificant error in determination of the repair timings within the used measurement intervals of 6 h^28,34,41^.

DNA DSBs dynamics in U87 human glioblastoma cells was estimated using the comet assay. The peak number of DSBs was observed 18 h after exposure to MMC (*P* < 0.001) (**Figure 1E**). This pattern differed from that obtained for the primary cell culture^33^; thus, an independent therapeutic scheme for treating U87 cells based on the new data regarding the repair of MMC-induced ICLs has been developed (**Figure 1F**).

To determine the time of accumulation of the majority of tumor cells in the appropriate cell cycle phase, when they are most susceptible to the final treatment, two assays were performed. First, we have assessed the distribution of U87 cells along the cell cycle both during and after treatments with MMC; second, we have estimated the changes in TAMRA+ cells content along the course of treatments.

#### Distribution of U87 cells along the cell cycle both during and after treatments with MMC

Treatments with MMC were scheduled to achieve the “overlapping” cell cycle arrests, when each subsequent administration occurs directly prior to the end of DNA repair induced by the previous one.

**Figure 2:**
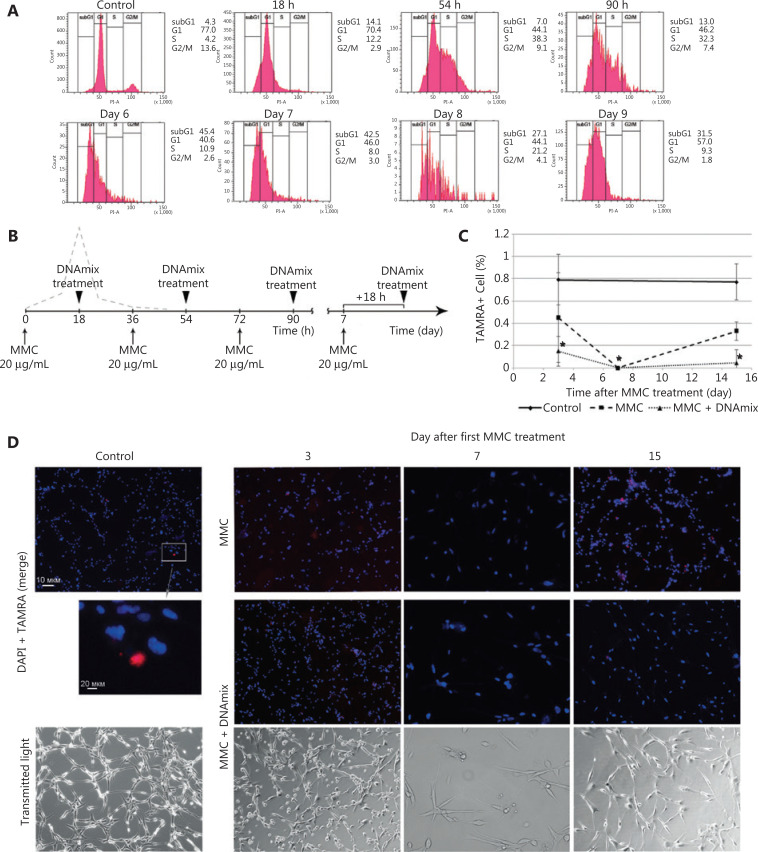
Basic Karanahan approach parameters and treatment schedule. (A) Results of the cell cycle assays in U87 cells prior to the treatment with MMC, 18, 54 and 90 h later as well as on days 6–9 after the first treatment. G1, S, and G2/M, phases of the cell cycle; subG1, apoptotic (presumably) cells. (B) Principle therapeutic scheme for treating U87 glioblastoma cells *in vitro*. Arrows indicate the appropriate timings for administration of cytostatic agent and composite DNAmix preparation. The dashed line displays the concomitant number of DSBs induced by the exposure of U87 cells to a cytostatic agent. (C) Percentage of TAMRA+ cells in U87 cell culture after treatments with MMC and DNAmix preparation according to the Karanahan approach. The average value ± 99% CI are given; confidence was estimated relative to the control using the Mann–Whitney criterion in the Statistica 10 software package, *P < 0.05. (D) Cytological preparations of U87 cells before (control) and on days 3, 7, and 15 since the start of therapy. The figure represents the merged image of DAPI (chromatin) and TAMRA (exogenous DNA) staining. For the control and MMC + DNAmix groups, images of cells in transmitting light are also given. MMC, group treated with cytostatic only; MMC + DNAmix, group treated with cytostatic in combination with DNA-based preparation.

Distribution of cells along the cell cycle was assessed by staining with propidium iodide followed be the FACS assay. Intact U87 cells demonstrated the “classic” distribution with two distinct peaks corresponding to G1 phase (2N set of chromosomes) and G2/M phases (4N set of chromosomes) and a “bridge” corresponding to S phase (**Figure 2A**, control). Exposure of these cells to a cytostatic compound induces two coinciding processes. The first is the significant slowing down in the replication of DNA in cells moving “forward” along the cell cycle, while the second is the “backward” flow of cells undergoing apoptosis, which is known to be tightly associated with DNA degradation. Being based on detecting the absolute amount of nucleic acid, which can be identical both in living and in apoptotic cells, the approach used does not allow distinguishing between these two processes and therefore provides a misrepresented picture of the cell cycle distribution, and the more intense these counterflowing processes, the greater such a misrepresentation. In this regard, after analyzing the cell cycle in treated cells, we have tried to consider only those experimental points, which implied the minimal degree of misinterpretation. These were either accumulation of cells in G2/M or transition from the G2/M peak into the G1 zone as well as more or less synchronous entry into the S phase.

Since the cell cycle assay implies the aforementioned misrepresentation and consequent limitations, we, in our studies, always assess the content of TAMRA+ cells as a marker indicating the efficacy of treatments applied against CSCs. Superimposing the results of these two approaches allows the most precise determining the time for the terminal therapeutic strike.

After triple exposure to MMC, U87 cells were monitored for their cell cycle distribution to determine the time for the final treatment with cytostatic compound, when the majority of cells were most susceptible to such a treatment (preferably in the G1 phase immediately prior to entering the S phase). The treatment scheme is shown in **Figure 1F**. As we previously demonstrated, triple exposure of tumor cells to cytostatic compounds induces the large-scale apoptosis in committed descendants that is detected microscopically clear. The rest of the cells, including TAMRA+ CSCs, undergo some sort of synchronization and then, on a certain day after exposures, more or less synchronously enter the S phase^28,34^.

As it can be noted from **Figure 2A**, the following patterns of U87 cell cycle distribution were observed. Severe depletion of cells from the G2/M occurs, and the majority of cells are accumulated in G1 after the initial exposure to MMC 18 h. At hours 54 and 90, a significant fraction (up to 50%) of cells is detected in the S and G2/M phases (massive cell cycle shift). Days 6 and 7 are associated with the drastic depletion of cells from both S and G2/M phases and their accumulation in subG1/G1 fraction, which indicates the ongoing large-scale apoptotic process that affects mainly the bulk of committed U87 tumor cells.

On day 8, the subG1/G1 fraction is getting smaller with the simultaneous growth of the S phase fraction (21% *vs.* 8% on day 7), which indicates the more or less synchronous entry of cells into dividing state. Based on these results, we have chosen day 7, which precedes the day of massive exit of cells into the S phase, as the most suitable for the terminal treatment with cytostatic agent. The principle therapy schematic is shown in **Figure 2B**.

#### Estimating the changes in TAMRA+/CD133+ U87 CSCs content during the course of treatments according to the proposed therapeutic schema

To validate the appropriateness of the previously determined day for the terminal “therapeutic strike”, we have used cytological assays to regularly monitor U87 cells throughout the whole course of *in vitro* exposure to MMC. Changes in the content of TAMRA+ cells upon introduction of DNAmix treatments into the therapy were also estimated.

Adherent U87 cells were *in vitro* treated either with MMC or with a combination of MMC and DNAmix preparation (see Materials and methods) in accordance with the schematic in **Figure 2B**. The percentage of TAMRA+ cells was estimated by direct counting the cells on cytological preparations on days 3, 7, and 15 since the start of treatments; total three samples were assayed for each experimental point (**Figure 2C and 2D**). Additionally, one of specimens from each experimental point was used for counting the absolute number of viable (adherent) cells and, in particular, TAMRA+ ones. In all cases, the counting was performed after assessing the relative content of TAMRA+ cells.

As it follows from **Figure 2C**, the content of TAMRA+ in U87 cell culture along this experiment was 0.79% ± 0.17%, and no reliable changes were found. On day 7 since the start of treatments, the percentage of TAMRA+ cells in both groups fell to 0% (within the assay sensitivity threshold) and began to grow slowly. On day 15, the MMC group had 0.34% TAMRA+ cells and MMC + DNAmix group had 0.1% TAMRA+ cells (*P* < 0.05 and *P* < 0.01, respectively).

As we mentioned above, one of the specimens used for assessing the relative content of TAMRA+ cells (**Figure 2C**) was also used for monitoring the changes in the absolute number of both viable cells in general and TAMRA+ cells in particular. Within 3 days, the total number of cells in the control specimen increased from the initial 3 × 10^5^ cells up to 5.7 × 10^5^ cells (1.9-fold). In the MMC and MMC + DNAmix groups, the number of viable cells gradually decreased: after 7 days, the number of cells was 142 × 10^3^ (2.1-fold decrease) and 94 × 10^3^ (3.2-fold decrease) respectively. After 15 days, viable cells accounted for 62 × 10^3^ (MMC) and 72 × 10^3^ cells (MMC + DNAmix).

During the experiment, the number of TAMRA+ cells in experimental groups decreased continuously: after 7 days, the minimum in their absolute number was detected; on day 15, these cells accounted for 210 (MMC) and 72 (MMC + DNAmix) cells of the initial 4.5 × 10^3^ cells.

It should be noted that on the 7th day since the start of treatments, the number of cells in specimens reached its minimum due to the apoptosis induced by these treatments, which was confirmed by FACS.

The data obtained evidence that the proposed therapeutic strategy provides a drastic decrease in quantity of TAMRA+ cells both upon the solitary use of MMC and especially upon a synergistic action of MMC and the DNAmix preparation. Moreover, these results confirmed day 7 to be critical for the subpopulation of TAMRA+ cells.

All the assays conducted evidenced that the proposed therapeutic regimen, namely the schedule of cytostatic agent and DNAmix preparation administration, as well as the day of terminal treatment, were determined correctly. Being applied, this approach provides an effective elimination of CSCs (TAMRA+ cells) upon a monotherapy with MMC and especially upon a combined treatment with MMC and composite DNAmix preparation. The following experiments on orthotopic grafting U87 cells, treated in full accordance with the Karanahan approach, proved the fact of specific elimination of CSCs as a source of tumorigenicity.

### Estimation of the tumorigenic potency of U87 human glioblastoma cells exposed to MMC and to MMC in combination with DNAmix

**Figure 3:**
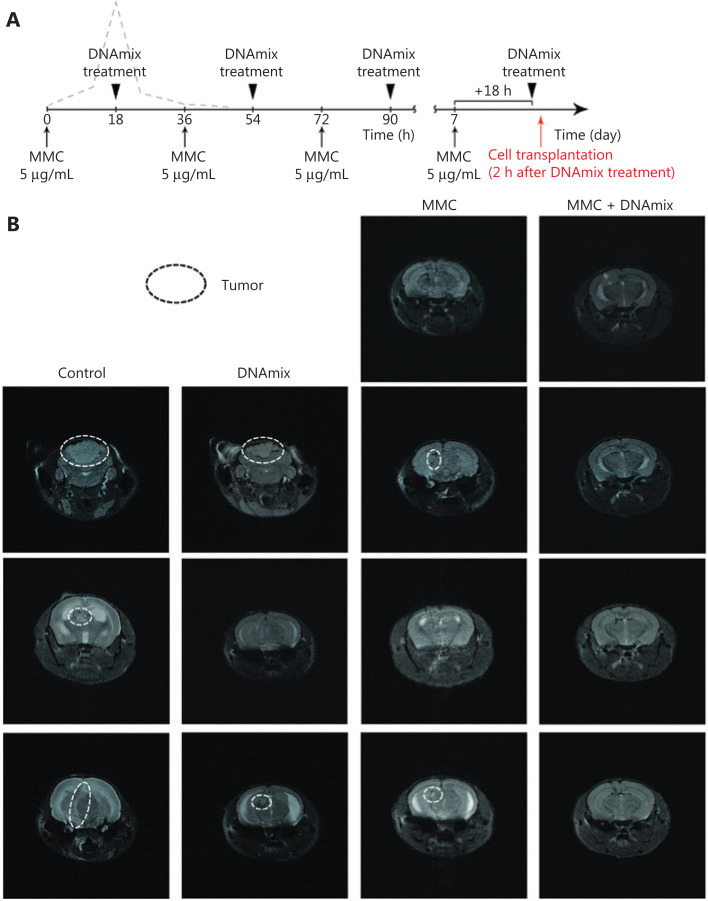
Intracerebral grafting of pretreated *in vitro* U87 glioblastoma cells into SCID mice. (A) Treatment schematic. (B) NMR tomography of the brain of SCID mice on day 24 after intracerebral inoculation of U87 human glioblastoma cells. Control, mice inoculated with untreated U87 cells; DNA, mice inoculated with U87 cells pretreated with DNAmix only; MMC, mice inoculated with U87 cells pretreated with MMC only; MMC + DNAmix, mice inoculated with U87 cells pretreated with MMC in combination with the composite dsDNA preparation.

U87 cells pretreated *in vitro* in accordance with the proposed therapeutic schema (**Figure 3A**) were intracerebrally grafted into immunodeficient mice. In preliminary experiments, upon the use of cytostatic in the dosage of 20 µg/mL, there was no noticeable difference observed upon the grafting of cells pretreated either with MMC solitary or with MMC in combination with DNAmix (data not shown). Additional experiments with a lower MMC dose of 5 µg/mL, which is closer to the therapeutic one^42,43^, revealed that the cellular response was identical for both cytostatic doses used. In this regard, in all further *in vitro* treatments of U87 cells, an MMC dose of 5 µg/mL was used.

Five groups of 3–4 mice each were formed: 1) the negative control group (injected with 5 µL of physiological saline solution, data not shown); 2) the positive control group (grafted with 3 × 10^5^ untreated U87 cells per mouse); 3) the DNAmix group (grafted with 4 × 10^5^ DNAmix-pretreated U87 cells per mouse); 4) the MMC group (grafted with 4 × 10^5^ MMC-pretreated U87 cells per mouse); 5) MMC + DNAmix group (grafted with 4 × 10^5^ MMC + DNAmix-pretreated U87 cells per mouse). NMR tomography images of the brain of grafted mice on day 24 after transplantation are shown in **Figure 3B**.

As it follows from tomography images, intracerebral grafts have developed both in mice inoculated with untreated U87 cells and in two of three mice inoculated with cells pretreated with DNAmix. In the case of negative control, grafts were not detected in all three animals in the group. Finally, in the group of MMC pretreated cells, grafts have developed in two of four animals (50%) only, whereas cells pretreated with MMC in combination with DNAmix produced no grafts at all. Thus, the proposed pretreatment of U87 glioblastoma cells with MMC in combination with the DNAmix preparation is proven to completely disrupt their tumorigenic entity, represented by the TAMRA+ cells.

### Approbation of the Karanahan approach for treating developed subcutaneous grafts of the human U87 glioblastoma in mice

In total, five full-scale experiments on subcutaneous engrafting U87 glioblastoma cells with following treatment of the resulting grafts in accordance with the proposed approach have been carried out, yielding fairly consistent results. The results of the latest one, in which all the previously faced problems, such as dissimilarity in grafts onset and early growth with consequent discrepancies in tumors values observed, are presented as an illustration.

**Figure 4:**
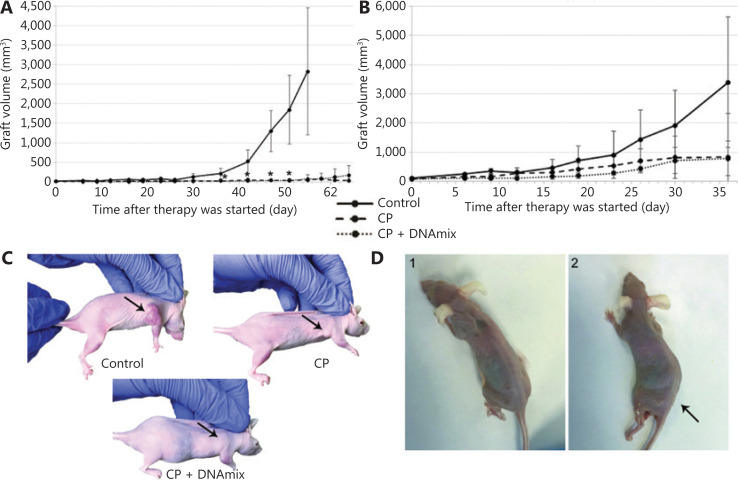
Therapeutic treatment of experimental mice with developed subcutaneous xenografts of U87 human glioblastoma. (A) Graft growth rates in mice with an average initial tumor size of 15 mm^3^ (*n* = 7 per group). (B) Graft growth rates in mice with the average initial tumor size of 92 mm^3^ (*n* = 6 per group). The median ± standard error of the mean are given; confidence was estimated using the Mann–Whitney criterion in the Statistica 10 software package, *P < 0.05. (C) Treated animals. (D) Animals from the CP + DNAmix group with a characteristic symptom complex manifestation: 1) animal with severe anorexia; 2) animal with hind limb paralysis (indicated by the arrow).

Mice with the average initial tumor size of 15 mm^3^ (**Figure 4A**) were treated separately from those with tumors of the average initial size equal to 92 mm^3^ (**Figure 4B**). The proposed therapeutic schema effectively inhibited the growth of subcutaneous grafts in both selected groups, and such a positive trend was observed both in mice receiving CP only and in those receiving a combined CP + DNAmix therapy (**Figure 4C**). In some similar experiments, such a combined CP + DNAmix therapy tended to produce a more pronounced inhibitory effect on grafts development than solitary CP treatments (data were not shown).

In the reported experiment in CP + DNAmix group, we have observed the so-called “window of death” manifestation described in our previous reports^30^. Two mice with the definitive absence of developed xenografts in the grafting site demonstrated the development of the characteristic symptom complex: severe anorexia (**Figure 4D1**) and paralysis of hind limbs (**Figure 4D2**). To clarify the cause of these disorders, animals were subjected to pathomorphological assays of internal organs (**Supplementary Figure S2**) and bone marrow components (**Supplementary Table S1 and S2, Supplementary Figure S3 and S4**). Results of these assays revealed that paralysis was due to encephalopathy, associated with a large-scale neuronic vacuolization and consequent degradation of neural cells^44^.

Examination of bone marrow samples indicated 1) a severe depletion of erythroid progeny with increased fraction of blast cells; 2) a noticeable increase in monocytic lineage fraction due to elevated number of mature monocytes; 3) catabolic changes in bone marrow cells with characteristic vacuolization of cytoplasm and nuclei, fragmentation of nuclei, appearance of granulated blast cells and cells with severely reduced cytoplasm, as well as other cellular abnormalities (**Supplementary Table S1 and S2, Supplementary Figure S3 and S4**). The figure shows degenerative processes in the hematopoietic system, characteristic of most severe forms of sepsis, abscesses, and acute liver dystrophy (found in pathomorphological examination).

### Approbation of the Karanahan approach for treating mice after surgical excision of the developed orthotopic grafts of the human U87 glioblastoma

This section shows the results of the experiments on the therapeutic treatment of the experimental U87 human glioblastoma with the Karanahan approach, applied after surgical excision of the orthotopic xenograft under conditions maximally close to clinical practice. In total, all procedures consisted of two main stages: 1) surgical excision of the developed orthotopic graft and 2) postsurgical therapy with cross-linking cytostatic agent CP in combination with the composite preparation of dsDNA (DNAmix), applied in accordance with preliminary determined biological parameters being specific for the tumor in question. The choice of cytostatic compound was due to the following reasons: 1) only cross-linking compounds cause the formation of ICLs, which activate recombinative events essential for the therapy to be effective (DNA-damaging effect and associated DNA repair mechanisms caused by TMZ are not described well and thus cannot be adapted for use within the Karanahan approach); 2) CP acts systemically; 3) approximately 20% of the acting CP metabolite, phosphoramide mustard, was shown to bypass the BBB^41^. In addition to the aforesaid, the surgical intervention as well as the tumor itself enhances the efficacy of delivering the active compound due to damaging the BBB.

The experiment has been carried out using 15 SCID-immunodeficient mice. All mice were subcortically injected with U87 cells (see Materials and methods) and further divided into three groups of five animals each: 1) the CP + DNAmix group, which postsurgically received the full therapy, namely CP in combination with composite dsDNA preparation; 2) the CP group, which postsurgically received a monotherapy with CP only; 3) the control group, which postsurgically received injections of physiological saline solution. The complete procedure of surgical tumor resection as well as postsurgical therapy are detailed in the Materials and methods section and in **Supplementary Figure S1**.

Of 15 animals, 12 were successfully operated on. The postoperative period proceeded without purulent-septic complications. Neurological deficit was also not observed. During the intraoperative phase, three mice died due to failure in blood circulation caused by hypothermia and aspiration of the stomach content into the respiratory tract because of anesthetic overdosage. Five days after surgical intervention, all mice were subjected to postoperative MRI. The radicality of tumor resection was estimated according to standard criteria: total removal (more than 95% of the tumor bulk removed), subtotal (80%–94% removed) and partial (50%–79% removed). **Table 1** represents the results of preoperative and postoperative volumetry of tumors.

**Table 1:** Preoperative and postoperative volumetry of tumors in mice

Group	Animal number	Preoperative tumor volumetry (mm^3^)	Postoperative tumor volumetry (mm^3^)	Radicality of tumor resection (%)
Cyclophosphamide + DNAmix	1_1	4.692	0	100*
	1_2	3.103	0	100*
	1_3	5.622	2.222	61^†^
	1_4	2.446	0	100*
Cyclophosphamide	2_1	5.662	0	100*
	2_2	2.818	2.718	–
	2_3	1.974	0	100*
	2_4	63.364	16.251	75^†^
Control	3_1	3.434	0	100*
	3_2	41.736	16.548	61^†^
	3_3	0.134	0.239	–
	3_5	5.106	0	100*

*Total resection. ^†^Partial resection. BBB, blood–brain barrier; CNS, central nervous system; CP, cyclophosphamide; CSC, cancer stem cell; DC, dendritic cell; dsDNA, double-stranded DNA; HR, homologous repair; ICL, interstrand cross-link; MDSC, myeloid-derived suppressor cell; MMC, mitomycin C; MOF, multiple organ failure; MTIC, monomethyl triazen imidazole carboxamide; NER, nucleotide excision repair; TM, tail moment; TMZ, temozolomide.

Thus, tumors were resected totally in three of the four mice in the CP + DNAmix group and in two of the three animals in both the CP and control groups.

**Figure 5:**
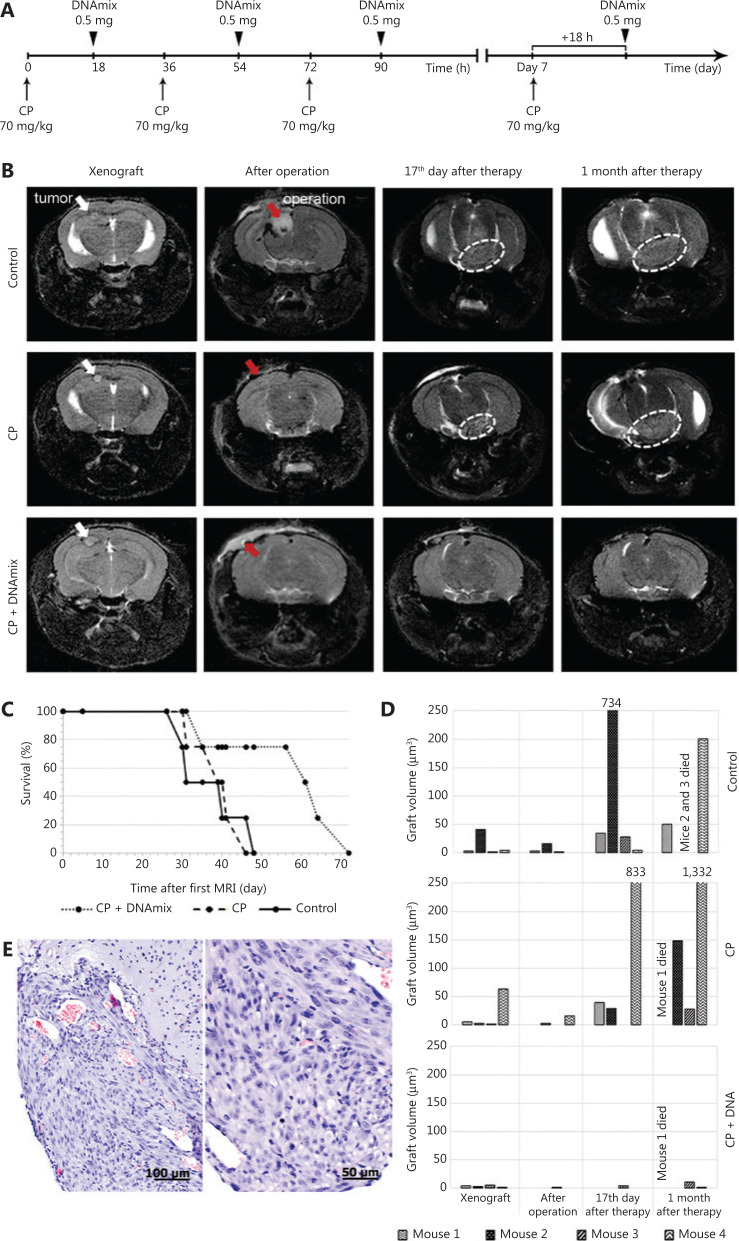
Magnetic resonance images (MRIs) of tumor nodes and survival rates of mice. (A) Schematic Karanahan approach with the schedule and dosages of CP and DNAmix administration. (B) Pictures of four sequential MRIs taken during the experiment. Preoperative MRIs indicate the presence of individual neoplastic formations with clear boundaries localized subcortically in the projection of parietal lobes (marked with white arrows) in all three groups. Postoperative MRIs indicate the total tumor resection (100% removal of tumor bulk) achieved in all the cases presented (areas of surgical intervention are marked with red arrows). On day 17 after the therapy is finished, MRIs indicate a dissemination of tumors through the cerebrospinal liquor paths to the brain basal zones (encircled with white dotted lines) in the CP and control groups. Thirty days since the end of therapy, a noticeable increase in tumor volume associated with infiltrative growth was observed in the CP and control groups, whereas the CP + DNAmix group displayed no signs of relapse. (C) Mice survival rates (Kaplan–Meier curves) during the experiment. Preoperative MRI is considered the start of the experiment. (D) Growth rates of tumors in individual animals by the experimental groups: control, CP, and CP + DNAmix. (E) Histological examination of the relapsing tumor from a mouse in the CP + DNAmix group.

The Karanahan approach was applied in accordance with exactly the same schema used in mice with subcutaneous grafts (**Figure 5A**), with one exception: in this case, DNAmix was administered subcutaneously in the area of craniotomy window instead of intratumoral injections in the case of subcutaneous grafts. In addition to preoperative and postoperative MRIs, these examinations were also carried out on days 17 and 30 after the therapy finished (**Figure 5B**).

Seventeen days after the treatment, 75% of animals in the CP + DNAmix group displayed no signs of growing tumors, whereas in the control and CP groups, tumors disseminated to the brain basal zones through the cerebrospinal liquor paths. Thirty days after the treatment, one mouse in the CP + DNAmix group (25%) has died, another one (25%) displayed no signs of growing tumor, and the 2 remaining mice (50%) had bulky formations observed in the projections of primary tumor localization, but the volumetric data indicated a significantly slower growth rate compared with the other groups (CP and control), in which infiltrative tumor growth was detected. As it follows from the results of NMR tomography and estimation of survival spans, the Karanahan approach drastically slowed down the tumor growth and increased the mice survival median, which was 62 days in the CP + DNAmix group being twice longer than in the control group (*P* = 0.1 by the Mann–Whitney criterion) (**Figure 5C and 5D**). In the CP group, this parameter was 39 days producing no significant differences (*P* = 0.46) relative to the control group (**Figure 5C**). Pathomorphological examination of the brain sample of the mouse with recurrent tumor growth from the group CP + DNAmix confirmed that the recurrent neoplasm had the histological characteristics similar to those of U87 glioblastoma (**Figure 5E**).

## Discussion

The main objective of the reported investigation was to adapt the principles of the proposed the Karanahan or 3 + 1 anti-tumor therapeutic approach to the real clinical practice. At the first stage, in absolute accordance with the aforementioned principles, the basic properties of U87 cells essential for the effective eradication of CSCs were determined. Based on these findings, we have developed the universal therapeutic routine, which was further used for treating U87 cells both *in vitro* and *in vivo* in either form of xenografts (ectotopic and orthotopic). The results obtained indicate the high therapeutic potential of the proposed approach in treating human glioblastomas.

In our early experiments, we have demonstrated that the main factor disrupting the grafting capabilities of cell lines is not the immune response, but the loss of tumorigenic potential due to elimination of TAMRA+ CSCs from the cellular population during *in vitro* pretreatments, as confirmed in **Figure 3**^26,28,29,34^. It is this factor that was presumed to be critical for the successful therapy in experiments on treating ectopic subcutaneous and orthotopic intracerebral xenografts of U87 cells in mice (**Figures 4 and 5**).

The experiments conducted once again demonstrated that the synergistic action of CP with high doses of DNAmix (0.5 mg per mouse administered parenterally/intratumorally) is extremely toxic.

In our previous investigations, we have found that mice with engrafted Krebs-2 that are treated with dsDNA in a certain time span after CP administration (denoted as “death window”) die of the specific symptom complex. Further investigations revealed two equally possible causes for this phenomenon. In the first case, injection of dsDNA within the certain time span(s) during the process of repairing CP-induced ICLs provokes the depletion of lymphoid stem in the red bone marrow, and it takes a month to restore^30^. As a result of developed immunodeficiency, activated pathogenic microflora provokes the development of systemic inflammation, multiple organ failure (MOF) and consequent death of animals. In the second case, injection of a large amount of dsDNA fragments, which are classic damage-associated molecular patterns, induces the microvascular thrombosis and, once again, systemic inflammation enhanced by the necrosis of lymphocytes that also results in MOF and death of animals^27,45^.

To clarify the causes of this pathology phenomenon observed in experiments with U87 glioblastoma, a pathomorphological examination of organs and tissues from two mice with a pronounced symptom complex has been performed.

One mouse demonstrated the terminal stage of anorexia, whereas the other one developed hind limb paralysis (Supplementary data). During the examination, destructive neuronal changes were detected in both mice. Cytoplasmic vacuolization of neurons and neuropil were observed, indicating a severe cellular dystrophy, which is mostly typical of prion pathologies^46,47^. Bone marrow cell examination also revealed large-scale degenerative changes. Pronounced monocytosis, which is typical of acute and chronic infections as well as of developing malignancies of various localizations, was detected. Cytological assays revealed an almost complete palette of pathological changes, including the pronounced vacuolization of cytoplasm, granularity in blast cells, “foamed”, fragmented, and vacuolized nuclei, cells with doubled nuclei, with bubbling cytoplasm or without cytoplasm at all. In general, the picture observed is highly typical of the most severe forms of sepsis, abscess, and acute liver dystrophy.

Another characteristic peculiarity of mice with developed symptom complex was a complete reduction of developed xenograft, which could not be detected at the grafting site on the moment of pathomorphological examination. At the same time, tumor cells were found to be disseminated across the organism and detected in a variety of organs and tissues. It could be presumed that therapeutic intervention with CP + DNAmix somehow disrupts the integrity of cellular and/or stromal structure of the grafts, and this effect could be associated with the elimination of tumor-organizing component represented by CSCs, as it was described previously for the sphere-organizing structure^35,48^. As a result, committed tumor cells, deprived of their tumor-initiating entity, chaotically disseminated across the organism, becoming vulnerable to the immune system as shown for Krebs-2 carcinoma^28^.

At this, there is an obvious risk of incomplete elimination of CSCs due to objective problems with delivering DNAmix to the targets upon its intratumoral or any other form of administration. In this case, the residual CSCs (most probably those, which are disseminated across the organism) will inevitably develop a recurrent growth. Upon dissemination of committed tumor cells, only transient, non-aggressive, or weakly aggressive formations, which develop due to residual proliferative capabilities of the committed progeny and degrade in short time, will be observed (as it was shown for the other tumor model of murine Lewis carcinoma). In this case, two scenarios can be presumed. 1) Suspecting a malignant dissemination after removing the tumor burden due to the applied Karanahan approach, it is supposed to use “traditional” drugs aimed at CSCs, which, being deprived of their native microenvironment, become quite vulnerable to such drugs. 2) In the case of non-malignant dissemination, the Karanahan approach with DNAmix excluded from the scheme should be reapplied, as we have done in other our work (data currently in progress). The use of CP in monotherapy with precisely determined timing can also result in effective eradication of CSCs and complete cure of mice with developed Krebs-2 carcinoma^29^. In fact, we believe that any of those current therapy used in cancer treatment will be more effective after a course of the Karanahan approach.

There is another objective problem in the implementation of the Karanahan approach that is related to the possible overlap of DNA repair processes in CSCs and normal hematopoietic cells. If the timings of these processes in both types of cells coincide, then the elimination of CSCs will coincide with the appropriate symptom complex we observe within the “death window”. In the mouse models we have used, the process of DNA repair in the investigated tumor cell lines did not overlap with that in normal hematopoietic cells that allowed using the Karanahan approach with no limitations^26^. If such an overlap is detected in clinical settings, the symptom complex of “death window” is to be resolved by the reinfusion of preserved bone marrow hematopoietic cells taken before the start of therapy^28,49^. Another possible approach is the use of antibiotics (or any other affordable ways) for suppressing opportunistic infections during the time span required for the restoration of functional immune response^45^. Therefore, the results obtained suppose the development of additional procedures aimed at resolving the “window of death” symptom complex appearing during the proposed therapeutic approach.

Metronomic injections of CP according to the determined time frames is the mandatory condition of successful implementation of the described approach. Inclusion of DNAmix into therapeutic procedures enhances the efficacy of CSCs elimination, but is not decisive. In this regard, another approach to treating malignant neoplasms (including experimental glioblastomas) based on metronomic administration of low/moderate doses of CP in combination with TMZ, the main therapeutic drug used for treating glioblastomas, may be of interest. Doses and timings of cytostatic administration within the frames of CP low/moderate doses chemotherapy are close to those of the Karanahan approach that in some approximation allows reckoning such chemotherapy a variant or part of the Karanahan approach. Such a comparison can allow the re-evaluation of the possible role of CP as well as the Karanahan approach (as a complex therapy with low/moderate doses of CP) in treating glioblastomas.

TMZ belongs to the cohort of alkylating cytostatic agents that undergo *in vivo* metabolic conversion into acting compounds. In the case of TMZ, such an acting compound is monomethyl triazen imidazol carboxamide (MTIC), which induces the formation of three methylated nucleobase adducts: O^6^mG, N^3^mA, and N^7^mG. MTIC easily bypasses the BBB that makes it the main therapeutic agent in treating glioblastomas. Being used in therapeutic dosages, TMZ exerts its cytotoxic effect that results in structural chromatin disorders and consequent apoptosis of actively proliferating tumor cells.

Currently, one of the most “spotlighted” approaches in treating experimental malignancies is metronomic low-dose cytostatic administration^50^, and CP is proven to be the most effective in such metronomic low-dose cytostatic administration.

CP and TMZ were compared in terms of their effectiveness against ectopic and orthotopic xenografts of the following experimental tumors: 4T1 (mammary gland tumor), Panc02 (pancreatic tumor), and CT26 (colorectal cancer). TMZ, even in combination with CpG (activator of dendritic cells), turned out to be drastically less effective in low-dose metronomic therapy than CP^51^. Thus, it was demonstrated that low-dosage metronomic chemotherapy with CP caused a strong reduction of ectopic xenografts of Gl261 human glioblastoma, while TMZ produced rather weak anti-tumor effect in these experiments^52,53^.

Meanwhile, other investigators obtained exactly the opposite results in the same experimental model. In this case, TMZ, being administered in low doses on days 0–6–12 (schedule that was previously established for CP), produced a much more pronounced effect relative to CP, which also affected the Gl261 orthotopic graft growth, but at a definitely lower degree^54^.

The anti-tumor effect of metronomic low-dosage CP administration is believed to be associated with 1) remodeling the “pro-tumor” properties of myeloid suppressors and, in particular, reversal polarization of tumor-associated macrophages in combination with Tregs deprivation and 2) the unique capability of CP to activate immune cells, particularly those infiltrating the tumor, such as dendritic cells (DCs), natural killer, and natural killer T cells^51–53,55–60^. Since only 20% of phosphoramide mustard, the acting CP metabolite, reach the brain^41^, and its immune-stimulating effect in the case of intracerebral grafts is obviously lower. Moreover, BBB also impedes the migration of activated immune cells from peripheral blood to the tumor node. It was shown that the time necessary to repair the CP-induced ICLs does not exceed 36 h^33^, which means that, on days 6–7 (**Figure 2A**; on days 6–7, cells are synchronized and accumulated in the subG1/G1 phases of the cell cycle, and the 7th day was chosen for the next scheduled CP administration), all DNA repair processes are completed and cells are “ready” for the next treatment. Moreover, low doses of ICL-inducing compound allow a significant fraction of cells to repair all lesions quite correctly and, as a result, to retain their functional properties and even acquire resistance to the compound used.

In contrast to CP, TMZ easily bypasses the BBB and reaches the target – intracerebrally localized glioblastoma cells. Cytostatic effect of TMZ induces the appearance of tumor antigens, but the involvement of immune system in TMZ action mechanics is being disputed. Cytoreducing effect of TMZ mainly targets actively proliferating cells, but immature monocytes, DCs, and macrophages are known to be also affected^61,62^. It results from the peculiarities of the methylated nucleobase repair process, which is associated with the formation single-stranded breaks, which, in turn, can result in DSBs if such methylated nucleobases are located in both strands in the immediate vicinity to one another. Immature monocytes, in contrast to mature DCs and macrophages, turned out to be incapable of complete repairing such lesions and undergo apoptosis that results in gradual depletion of their progeny – DCs and mature macrophages. In this case, TMZ exerts the definitive immunosuppressive effect aimed against the normal myeloid progenitors. Meanwhile, depletion of tumor-infiltrating myeloid-derived suppressor cells (MDSC)^63^, which are also a progeny of immature monocytes, would produce an opposite, immunostimulating effect.

All antigens that appear in the brain are known to be immediately drained into the cervical lymph nodes, where intracerebrally localized DCs also migrate. Both intracerebrally primed DCs and those primed in lymph nodes induce the proliferation of adaptive T8+ lymphocytes. Some of these cells further return into the brain. Thereby, the large-scale adaptive immune response develops in CNS^61,62^.

It can be presumed that DCs that are primed by glioma antigens and completed DNA repair and that retained their functionality after exposure to TMZ induce immune response development. Macrophages that completed DNA repair and retained their functionality after exposure to TMZ lyse tumor cells, while tumor-infiltrating MDSCs are being eliminated. As a result, the cytoreducing effect of TMZ is enhanced.

The aforementioned information means that, with equal dosages in the node of exposure, CP produces a definitely higher immunostimulating effect relative to TMZ. Meanwhile, in the case of intracerebral tumors, when the issue of drug delivery begins to play a decisive role, it is TMZ that provides a greater anti-tumor effect.

The difference in duration of repairing ICLs and methylated nucleobases can be proposed as another explanation for the greater efficacy of TMZ relative to CP in regard to orthotopic xenografts. In the current report, we have demonstrated that U87 cells completely repair ICLs within 36 h, and to arrest these cells for a longer period, triple sequential exposure to CP is required. It is the only way to arrest the cells in question for 6 days using CP, after which they synchronously enter the cell cycle and accumulate in the G2/M phase. In contradistinction to CP, single exposure to TMZ arrests U87 cells for up to 72 h, when approximately 60%–65% of these cells are in the G2/M phase^28,64^. It can be presumed that in the aforementioned model^54^, TMZ caused the synchronization of cells exactly on day 6, when the next exposure provided either complete or substantial elimination of CSCs. In the case of CP, due to other DNA repair mechanics and associated time requirements, the necessary synchronization does not occur, and as a result, all the described CP-mediated anti-tumor effects, providing the effective elimination of CSCs, do not appear.

Considering all the above, it can be supposed that all the differences observed upon the metronomic low-dosage administration of CP and TMZ could be due to different mechanisms of their action toward the grafts of different origins and localizations.

The investigation conducted implies additional therapeutic capabilities of using CP in treating glioblastomas, and an example of such new capability is the synergistic action of CP and complex composite DNA-based preparation within the frames of the currently reported Karanahan approach.

## Conclusions

Results indicate that treatment of the experimental U87 glioblastoma in accordance with the Karanahan approach provides an effective elimination of TAMRA+ CSCs and thus could make the principle for a new approach in curing glioblastomas.
