# Supplementary material for: Efficacy of the new therapeutic approach in curing malignant neoplasms on the model of human glioblastoma
Source: Cancer Biol Med. 2021 Aug 15;18(3):910–30. doi: 10.20892/j.issn.2095-3941.2020.0511 (PMC8330538; doi:10.20892/j.issn.2095-3941.2020.0511)
Supplement: Supplementary file 1 [file cbm-18-910-s001.pdf]

Supplementary materials

Neurosurgical excision of the developed tumor

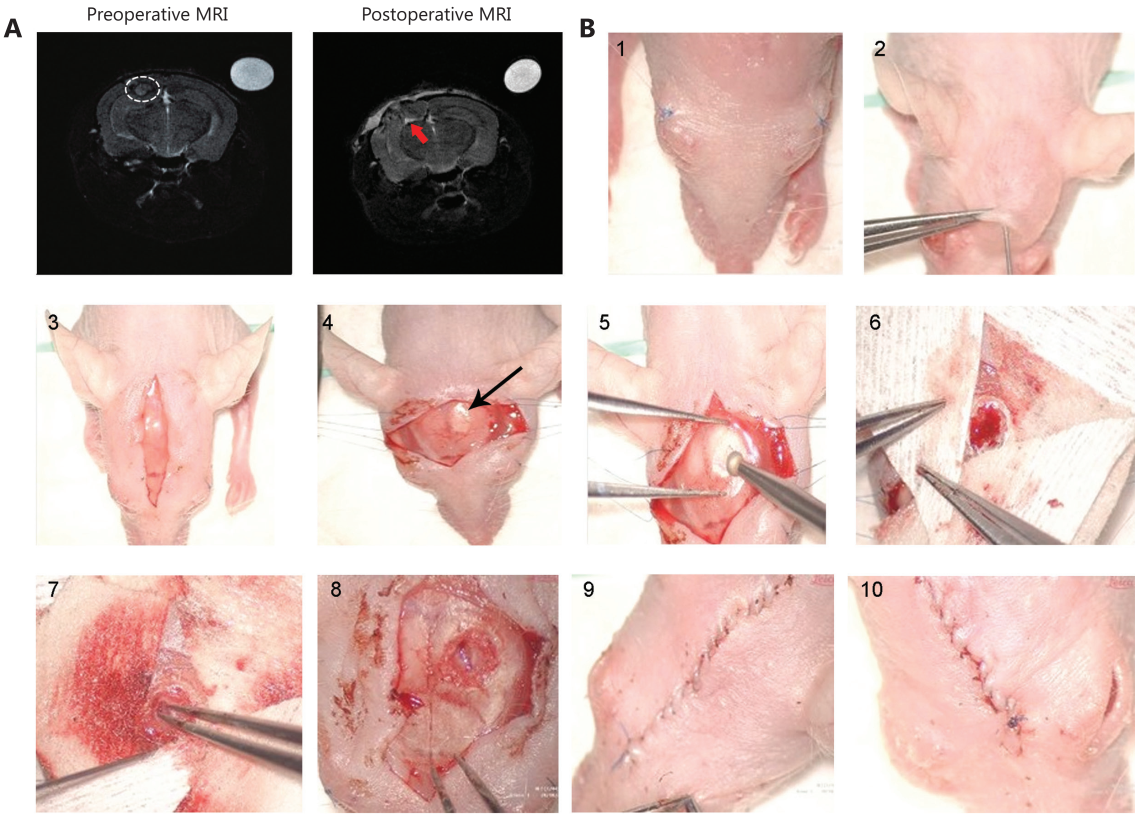

Figure S1 Sequential steps of the neurosurgical excision of developed U87 intracerebral xenografts in SCID mice. (A) Preoperative (left) and postoperative (right) T2-weighted technique images. The residual tumor volume is still detectable (right image). (B) Sequence of surgical stages. 1) Blepharorrhaphy: single nodular sutures were applied onto eyelids; skin was treated with an alcohol solution of chlorhexidine. 2) Subcutaneous administration of a 1% Novocaine solution. 3) Linear incision of the skin. 4) Fixing the scalp with holding stitches; developed graft (white, denoted with an arrow) is visible in the left parietal region. 5) Craniotomy stage (drill head diameter, 2 mm). 6) As soon as the tumor was accessed, the surgical wound was surrounded with sterile covers moistened with physiological saline solution. 7) Tumor excision step: a corticotomy was performed with an insulin syringe; tumor fragmentation and removal were performed with tweezers and suction by the syringe during the piston retraction. 8) General appearance of the surgical wound after removal of the tumor bulk. 9) The wound is sutured with a continuous twining seam using 6/0 Vicryl; sutures and surgical wound area are treated with an alcohol solution of chlorhexidine. 10) Stitches imposed during blepharorrhaphy are removed.

Pathomorphological examination of the internal organs of SCID mice after therapeutic treatment according to the Karanahan approach

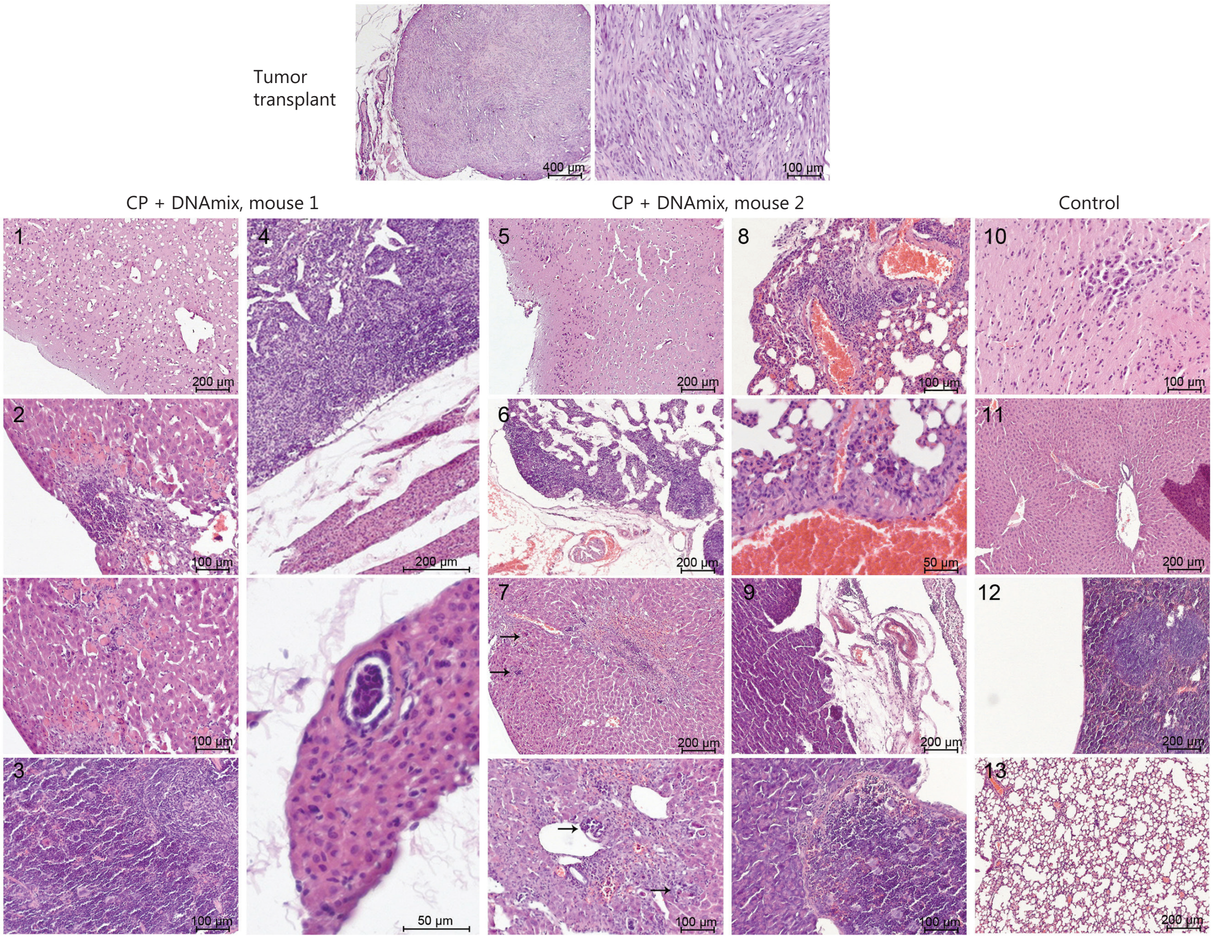

Figure S2 Pathomorphological examination of the internal organs of SCID mice with severe anorexia (mouse 1) and paralysis of hind limbs (mouse 2) after CP + DNAmix therapy and a control animal with U87 xenograft. Tumor transplant. Predominantly spindle-shaped cells are clearly seen. CP + DNAmix mouse 1. Pronounced vacuolization of neurons and neuropil determined in the cerebral cortex. In lungs, nodes of atelectasis and edema of the interstitial tissue as well as rare cells with morphological attributes of atypical xenograft elements are detected. In the liver, multiple metastatic nodes, polymorphic cellular infiltration, and necrosis in different parts of the lobules are observed across the entire section area. The red pulp zone of the spleen is filled with tumor cells; no signs of inflammation were found. The capsule of the tumor node is indistinct, conglomerate of spherical shape; polymorphic spindle-shaped cells form cords intertwining in different directions. Tumor tissue contains multiple blood vessels and cribriform areas (vacuolized fragments of tumor tissue). In the lymph node, atypical cells are observed both in the reticular tissue of the node itself and in the perifocal tissue as well as in the perivascular area. The lumens of some vessels are clogged with clusters of tumor cells. 1) Pronounced vacuolization of neurons and neuropil in the cerebral cortex. 2) Necrotic focus in the site of tumor cell infiltration in the liver; intact normal parenchyma is almost completely replaced by the large necrotic foci. 3) Red pulp zone of the spleen filled with tumor cells. 4) Lymph node with perifocal bundles of muscle fibers; both the node itself and muscle fibers are predominantly replaced by metastatic tumor cells; in the lower picture, a blood vessel clogged with atypical cells can be observed. CP + DNA mouse 2. In the cortex and white matter of the cerebral hemispheres, small foci of round cell infiltration are detected. In the lungs, pronounced edema and disinflation of parenchyma are found; sporadic perivascular and intraluminal aggregations of atypical xenograft cells are detected; inflammatory reaction is weak, but small amounts of neutrophilic granulocytes and lymphoid cells can be observed in the stroma of alveolar septa. In the liver, multiple loci of tumor invasion, parenchyma necrosis, and pronounced inflammatory infiltration of a mixed composition with neutrophilic inclusions are detected. Visually, necrotic sites are not always associated with metastatic foci. Lymphoid follicles in the spleen are intact, but the red pulp is filled with multiple neutrophilic granulocytes; small necrotic sites among the aggregated neutrophils as well as

clusters of tumor cells are found. Rare tumor cells can be detected on the outer surface of the pancreatic capsule. Lymph node parenchyma is predominantly replaced by atypical cells. 5) Diffuse round-cell infiltration of the surface layers of the cortical substance in combination with moderate vacuolization of neurocytes. 6) Pronounced edema of the lymph node parenchyma and perinodal adipose tissue; infiltration of reticular stroma with tumor cells. 7) Large site of necrosis surrounded by the multiple accumulations of metastatic cells (denoted with arrows); closeup of several metastatic nodes (denoted with arrows) with pronounced polymorphic cellular infiltration. 8) Severe cellular inflammation in the basal region of the lung and accumulation of metastatic tumor cells; closeup of one of the atypical cell clusters from the previous picture. 9) Strong intraluminal and perivascular metastatic infiltration of the pancreas and adjacent tissues; the lower picture shows the commissure of the spleen and pancreas by the tumor tissue. Control. In the cerebral cortex, a number of polymorphic cells morphologically similar to those of the xenograft were found. In interalveolar septa of the lungs, small aggregations of atypical cells are present, but the general parenchyma structure appears intact; signs of cellular inflammation are almost undetectable. No pathological changes were found in the liver. In the spleen, white pulp follicles retained their integrity; small clusters of atypical cells of the xenograft are detected in the red pulp, no signs of inflammation can be visualized. 10) Cluster of polymorphic cells in the cerebral hemisphere cortex. 11) Liver: no signs of pathological changes. 12) Spleen: white pulp follicles in the center of the picture; uniform infiltration of the red pulp with small islets of tumor cells. 13) Lungs: moderate hyperemia at minimal degree of the cellular inflammation; no clear signs of metastatic process.

Comparative examination of bone marrow cells (BMCs) from SCID mice with U87 xenograft after therapeutic treatment (mouse 1, with anorexia; mouse 2; hind limb paralysis) and untreated control animal

Table S1 Composition of the bone marrow by the appropriate hematopoietic lineages (%)

	Control	Mouse 1, anorexia	Mouse 2, paralysis
Erythroid lineage			
Total	**18.88**	**17.73**	**14.64**
Pronormoblasts	1.41	4.28	7.27
Basophilic normoblasts	3.82	8.27	4.62
Polychromatic and orthochromatic normoblasts	13.65	5.17	2.75
Lymphoid lineage			
Total	**15.7**	**15.07**	**8.55**
Large lymphocytes	2.55	3.55	1.18
Medium and small lymphocytes	13.15	11.52	7.37
Monocyte lineage			
Total	**23.06**	**34.56**	**37.23**
Monoblasts	3.68	7.39	5.8
Monocytes	19.38	27.18	31.43
Granulocyte lineage			
Total	**41.94**	**32.64**	**39.59**
Myeloblasts and promyelocytes	1.41	0.44	1.28
Neutrophilic metamyelocytes and neutrophilic band cells	6.79	8.57	8.64
Segmented neutrophils	31.68	21.57	27.5
Eosinophils	2.05	2.07	2.16

Table S2 Composition of the non-viable BMC fraction in the investigated groups (%)

	Control	Mouse 1, anorexia	Mouse 2, paralysis
Viable cells	83.91	73.19	80.54
Non-viable cells	16.08	26.81	19.46
Among non-viable			
Identifiable	0	0	0
Erythropoietic cells			
Lymphocytes	0.3	3.83	4.98
Monocytes	0.83	1.79	1.98
Granulocytic series	0.24	3.75	1.66
Unidentifiable (smudge cells)	14.42	17.44	10.84

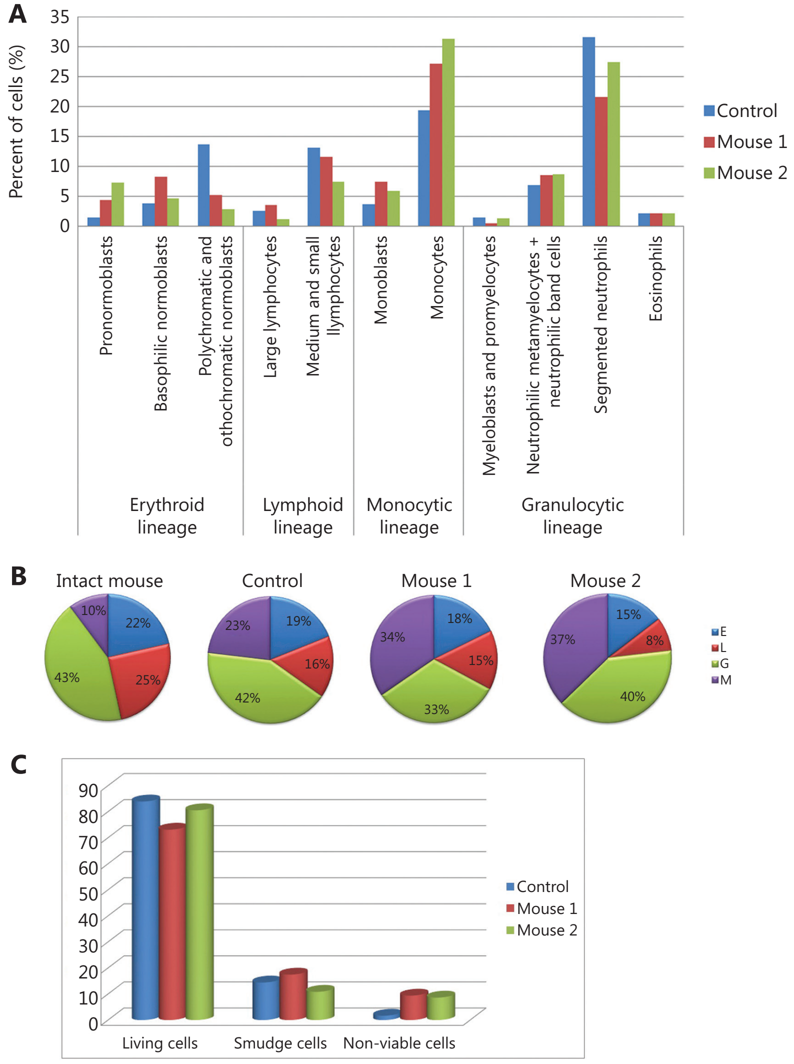

Figure S3 Comparative examination of BMCs from SCID mice with U87 xenograft after therapeutic treatment. (A) Cellular composition of the bone marrow in experimental animals. There is a depletion of erythroid cells: in animals with anorexia and paralysis, the number of blasts exceeds the norm, whereas the content of mature cells is noticeably decreased, especially in the animal with paralysis. The content of cells

of monocytic lineage is severely increased due to the fraction of mature monocytes. (B) BMC content (%) in different animals: in the control animal (untreated mouse with U87 xenograft), the content of monocytes is higher relative to the absolutely intact mouse, whereas in animals with anorexia and paralysis, it reaches its maximum. (C) Ratio of viable to non-viable cells in experimental animals: the number of viable cells is high in all cases; there are smudge cells (non-viable unidentifiable cells) in all preparations; some of non-viable cells can be related to certain hematopoietic germs, and their number in animals with anorexia and paralysis is increased relative to the control one.

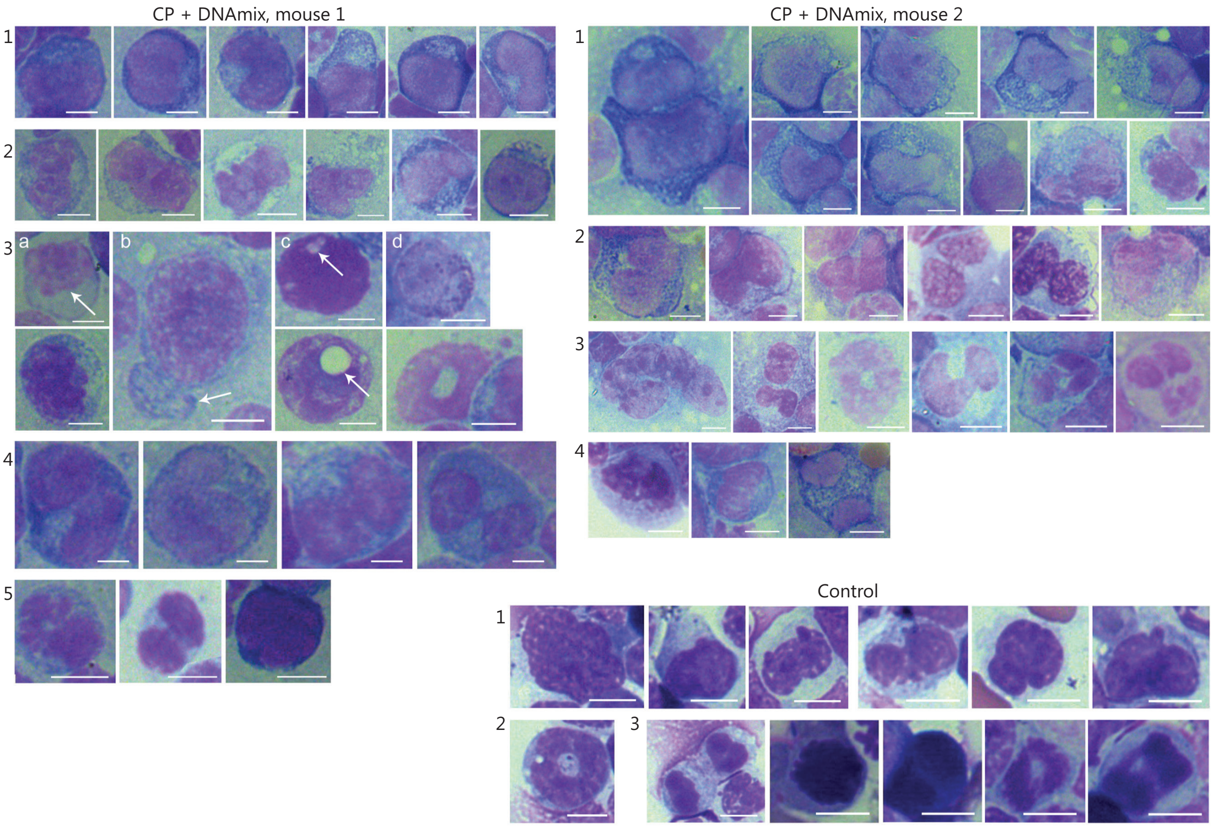

Figure S4 Pathomorphological changes of BMCs in mice with a characteristic symptom complex formed after the combined therapy with CP and DNAmix preparation according to the Karanahan approach. CP + DNAmix, mouse 1 (anorexia). 1) Large cells with nuclei of irregular shape and vacuolized and excessively granular cytoplasm. 2) Cells with excessively vacuolized cytoplasm. 3) Examples of non-viable cells: (a) with the "foamed" nucleus; (b) with the vacuolized cytoplasm; (c) with the vacuolized nucleus; (d) others. 4) Cells with doubled nuclei. 5) Mitosis-like formations. CP + DNA, mouse 2 (paralysis of both hind limbs). 1) Large cells with excessively vacuolized cytoplasm. 2) Cells with doubled nuclei. 3) Non-viable cells. 4) Mitosis-like formations. Control, non-treated mouse with the developed U87 xenograft. 1) Cells with nuclei of irregular shape. 2) A cell with the vacuolized nucleus. 3) Mitotic cells. Bars correspond to 5 μm.
